# Regulation of tocopherol (vitamin E) biosynthesis by abscisic acid-dependent and -independent pathways during abiotic stress in *Arabidopsis*

**DOI:** 10.1007/s00425-025-04670-9

**Published:** 2025-03-20

**Authors:** Victoria Kreszies, Nina Hoppe, Katharina Gutbrod, Peter Dörmann

**Affiliations:** 1 Institute of Molecular Physiology and Biotechnology of Plants (IMBIO), University of Bonn, 53115 Bonn, Germany; 2 Forest Botany and Tree Physiology, University of Göttingen, 37077 Göttingen, Germany

**Keywords:** Abscisic acid, *Arabidopsis thaliana*, Drought, High light, Osmotic stress, Nitrogen deprivation, Polyethylene glycol, Tocochromanol, Tocopherol

## Abstract

**Main conclusion:**

The increase in tocopherol (vitamin E) biosynthesis in *Arabidopsis* during drought and osmotic stress, but not during high light or nitrogen deprivation, is mediated by abscisic acid.

**Abstract:**

Plants increase the production of antioxidants including tocochromanols (vitamin E) during stress. To study the regulation of tocochromanol synthesis, *Arabidopsis* plants were exposed to drought, osmotic stress stimulated by polyethylene glycol, abscisic acid (ABA), nitrogen deprivation, and high light. ABA treatment resulted in increased contents of tocochromanols, and expression of the key tocopherol biosynthesis genes *VTE2* and *HPPD* was upregulated, indicating that tocochromanol accumulation was regulated by ABA. Under drought and osmotic stress, the ABA and tocochromanol contents as well as *VTE2* and *HPPD* expression were also increased. ABA levels did not change during nitrogen deprivation or high light treatment, indicating that tocochromanol accumulation under these conditions was ABA-independent. Tocochromanol accumulation during drought or osmotic stress was not compromised in the ABA-deficient *aba1-6*, *aba2-1* and *aba3-2* mutants, suggesting that tocochromanol synthesis under these conditions was mostly regulated in an ABA-independent way. Therefore, the accumulation of tocochromanols in *Arabidopsis* can be regulated by ABA-dependent and ABA-independent signaling pathways, based on the specific conditions.

**Supplementary Information:**

The online version contains supplementary material available at 10.1007/s00425-025-04670-9.

## Introduction

**Fig. 1:**
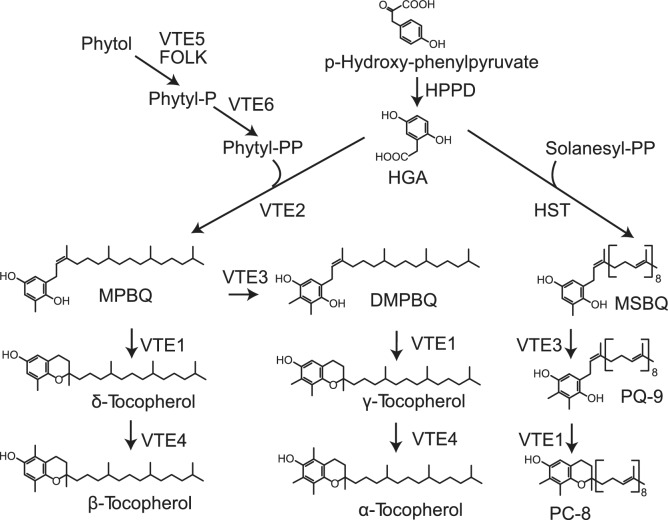
Tocopherol biosynthesis in *Arabidopsis*. Homogentisate is synthesized from p-hydroxyphenylpyruvate, a product of the chloroplast shikimate pathway. The side chain of tocopherol is derived from phytyl-diphosphate, which originates from phytol by two phosphorylation reactions. Two methylation reactions and a ring closure give rise to the different forms of tocopherol. Plastoquinol-9, which is synthesized from homogentisate and solanesyl-diphosphate, can be converted into plastochromanol-8 by VTE1. *DMPBQ* 2,3-dimethyl-5-phytyl-benzoquinol, *FOLK* farnesol kinase, *HGA* homogentisate, *HPPD* p-hydroxyphenylpyruvate dioxygenase, *MPBQ* 2-methyl-6-phytyl-benzoquinol, *MSBQ* 2-methyl-6-solanesyl-benzoquinol, *PC8* plastochromanol-8, *phytyl*-*P* phytyl-phosphate, *phytyl*-*PP* phytyl-diphosphate, *solanesyl*-*PP* solanesyl-diphosphate, *PQ*-*9* plastoquinol-9, *VTE1* tocopherol cyclase, *VTE2* homogentisate phytyltransferase, *VTE3* 2-methyl-6-phytyl-benzoquinol methyltransferase, *VTE4* γ-tocopherol methyltransferase, *VTE5* phytol kinase, *VTE6* phytyl-phosphate kinase

Tocochromanols are a group of prenylquinols (tocopherols, tocotrienols, and plastochromanol-8/PC8) which exert vitamin E function in humans and which are produced in plants, algae and cyanobacteria (Cahoon et al. 2003; Mène-Saffrané and DellaPenna 2010). In *Arabidopsis*, the group of tocochromanols encompasses four forms of tocopherols and plastochromanol-8 (PC8). The α, β, γ, and δ forms of tocopherols carry a phytyl side chain and vary in the number and positions of methyl groups on the chromanol ring. PC8 is derived from plastoquinol-9 and, therefore, carries a solanesyl side chain. In plants, tocochromanols are found in chloroplasts, where they accumulate in thylakoids and plastoglobules (Soll et al. 1985; Vidi et al. 2006). The total tocochromanol content and composition varies dependent on the species, tissue and developmental stage (Fryer 1992; Kruk and Strzałka 1995; Bréhélin et al. 2007). The precursor for the tocopherol head group is derived from the chloroplastic shikimate pathway. Hydroxyphenylpyruvate dioxygenase (HPPD) converts p-hydroxyphenylpyruvate into homogentisate (HGA) (Norris et al. 1998) (Fig. 1). The key step of tocopherol biosynthesis is the condensation of homogentisate with phytyl-diphosphate (phytyl-PP) catalyzed by homogentisate phytyltransferase (HPT, VTE2) (Collakova and DellaPenna 2003a). The 2-methyl-6-phytyl-1,4-benzoquinol (MPBQ) methyltransferase (VTE3) converts MPBQ into 2,3-dimethyl-6-phytyl-1,4-benzoquinone (DMPBQ) (Cheng et al. 2003). Next, tocopherol cyclase (VTE1) closes the second ring, producing δ-tocopherol and γ-tocopherol from MPBQ and DMPBQ, respectively (Porfirova et al. 2002). The last step is the methylation of δ-tocopherol and γ-tocopherol by γ-tocopherol methyltransferase (VTE4) yielding β- and α-tocopherol, respectively (Shintani and DellaPenna 1998). The characterization of two phytol kinase activities (VTE5, FOLK), which phosphorylate phytol, and phytyl-phosphate (phytyl-P) kinase (VTE6), which converts phytyl-P into phytyl-diphosphate (phytyl-PP), revealed that phytol for tocopherol synthesis is mostly derived from chlorophyll breakdown (Valentin et al. 2006; vom Dorp et al. 2015; Romer et al. 2024).

Water deficiency or drought represent one of the most limiting factors for plant growth (Boyer 1982). Other abiotic stresses including osmotic stress, nutrient deficiency, or excess light can limit plant productivity (Jenks 2007). During abiotic stress, reactive oxygen species (ROS) accumulate as byproducts of respiration and photosynthesis. Apart from their function as signaling molecules, ROS cause oxidative damage when their levels increase due to abiotic stress (Choudhury et al. 2017). Plants synthesize antioxidants including tocochromanols to scavenge ROS and to limit oxidative damage (Kobayashi and DellaPenna 2008). In line with this scenario, the tocochromanol content in leaves is increased under stress (Collakova and DellaPenna 2003b; Fleta-Soriano and Munné-Bosch 2017). The regulation of plant responses to abiotic stress is oftentimes mediated by phytohormones including abscisic acid (ABA) (Chen et al. 2020; Amanda et al. 2022). ABA is involved in the regulation of developmental processes such as embryo maturation, seed dormancy, germination, cell division, elongation and floral induction, as well as responses to environmental cues such as drought, salinity, cold and pathogen attack (Kumar et al. 2019). Abiotic stress stimulates ABA synthesis through induction of ABA biosynthetic genes, many of which harbor cis-regulatory elements in their promoters, known as ABA-responsive elements (ABREs) (Xiong and Zhu 2003; Shinozaki and Yamaguchi-Shinozaki 2007). Such ABA-specific motifs were also identified in the promoter regions of the tocopherol biosynthesis genes *OsHPPD*, *OsγTMT* and *OsMPBQMT1* in rice (Chaudhary and Khurana 2009), suggesting that tocopherol biosynthesis might be regulated by ABA. In addition, a positive correlation between the contents of ABA and of tocochromanols was found (Fleta-Soriano and Munné-Bosch 2017). Moreover, ABA-treated *Arabidopsis* seedlings contained increased levels of tocopherol, and expression of tocopherol biosynthesis genes was upregulated (Ghassemian et al. 2008).

ABA-deficient mutants of *Arabidopsis* (*aba*) which are characterized by reduced seed dormancy and increased tendency to wilt during water deficiency can be employed to study the role of ABA during the regulation of specific physiological pathways (Koornneef et al. 1982; Léon-Kloosterziel et al. 1996). The *aba1* mutant carries a mutation in the zeaxanthin epoxidase gene which results in the accumulation of zeaxanthin and reduced levels of ABA under stress (Xiong et al. 2002; Barrero et al. 2005). The *aba2* mutant carries a mutation in a short-chain dehydrogenase/reductase (SDR), causing a decrease in the conversion of xanthoxin into abscisic aldehyde (González-Guzmán et al. 2002). The final step of ABA biosynthesis is the oxidation of abscisic aldehyde to ABA, catalyzed by abscisic aldehyde oxidase (AAO3) (Seo et al. 2000). The *aba3* mutant is deficient in the molybdenum cofactor sulfurase (ABA3), which is responsible for the synthesis of the molybdenum cofactor required for the aldehyde oxidation (Léon-Kloosterziel et al. 1996; Bittner et al. 2001).

Here, we present the analysis of the regulation of tocochromanol biosynthesis during different abiotic stresses including drought, osmotic stress (polyethylene glycol 8000, PEG8000), direct ABA supplementation, N deficiency and high light stress in *Arabidopsis*. Drought stress and ABA supplementation resulted in strong ABA increases in the plants, while PEG8000 treatment caused moderate ABA increases. Nitrogen limitation or exposure to high light had a negligible effect on ABA contents. We furthermore analyzed the impact of ABA-deficiency on the accumulation of ABA and tocochromanols in leaves and seeds of *aba* mutants. From these studies, it became clear that tocochromanol synthesis is regulated by different mechanisms, including ABA-dependent and ABA-independent signaling pathways.

## Materials and methods

### Plants and growth conditions

The *aba* mutants of *Arabidopsis thaliana, aba1-6*, *aba2-1*, and *aba3-2,* and the ecotypes Col-0 and Ler were obtained from the Nottingham *Arabidopsis* Stock Centre (NASC). *Arabidopsis* seeds were surface-sterilized and sown on Murashige and Skoog (MS) medium (1 × MS basal salts, 2% sucrose, 10 mM MES-KOH, pH 6.0, 0.7% Phyto agar, Duchefa, Haarlem, Netherlands) (Murashige and Skoog 1962). After stratification at 4 ℃ for 24 h, the plants were incubated under a 16-h light/8-h dark light regime of 150 µmol m^−2^ s^−1^ and at 22 ℃ and 55% humidity.

For drought experiments, three seedlings each (grown on MS plates for 14 days) were transferred to one pot filled with 150 g of soil (Einheitserde Typ Topf 1.5, Gebrüder Patzer, Sinntal, Germany). The soil was kept well watered for another 14 days. Then, drought stress was initiated by withholding water for up to 10 days. Control plants were grown under well-watered conditions.

Osmotic stress was initiated with PEG8000 (Carl Roth, Karlsruhe, Germany). PEG8000 is heat-labile (van der Weele et al. 2000). Therefore, after autoclaving the medium (1 × MS basal salts, 2% sucrose, 10 mM MES-KOH, pH 6.0) in the presence of Gelrite agar (Duchefa; 0.3%, 0.6%, 0.8%, for control, 10% or 20% PEG8000, respectively), the medium was cooled to 80 °C, mixed with 0%, 10% or 20% PEG8000 (final concentration) and poured into Petri dishes (140 mm diameter). Ten to fifteen *Arabidopsis* plants (21 days old, raised on MS medium) were transferred to each plate, and leaves were harvested after 9 days.

For N deficiency experiments, plants grown for 2 weeks on MS plates were transferred to + N or –N medium and grown for another 14 days. The + N medium was composed of 0.7% Phyto agar, 1% sucrose, 2.5 mM KNO_3_, 1 mM MgSO_4_, 1 mM Ca(NO_3_)_2_, 1 mM KH_2_PO_4_, 1 mM NH_4_NO_3_, 25 µM Fe-EDTA, 35 µM H_3_BO_3_, 7 µM MnCl_2_, 0.25 µM CuSO_4_, 0.5 µM ZnSO_4_, 0.1 µM Na_2_MoO_4_, 5 µM NaCl and 5 nM CoCl_2_ (pH 6.0 with KOH). For nitrogen deprivation (-N), NH_4_NO_3_ was omitted, Ca(NO_3_)_2_ was replaced with CaCl_2_, and KNO_3_ was exchanged with KCl (Gaude et al. 2007).

For high light stress, 2-week old plants (grown on MS medium) were transferred to pots filled with soil and grown for another 3 weeks under well-watered conditions. Then, plants were exposed to high light of 500 µmol m^−2^ s^−1^ under a 16-h light/8-h dark cycle for 4 days or 8 days. Control plants were kept under normal light (150 µmol m^−2^ s^−1^) (Eugeni Piller et al. 2014).

After 3 weeks of growth on MS medium, 10–15 *Arabidopsis* plants were transferred to MS plates (140 mm diameter) containing 0, 10, 50, 100 or 150 µM of ( +)-*cis,trans*-abscisic acid (ABA; Olchemin, Olomouc, Czech Republic) and grown for another 9 days.

### Relative water content and water potential

The relative water content (RWC, in %) of *Arabidopsis* leaves was calculated according to the equation: RWC = (FW–DW)/(TW–DW) × 100, with FW, fresh weight of the leaf; TW, turgid weight (weight of leaf floated for 24 h in 5 mM CaCl_2_); DW, dry weight (weight of leaf after drying for 24 h at 60 ℃) (Sade et al. 2015). The water potential of solidified media was measured with the WP4C Water Potential Meter (METER Group, Munich, Germany).

### Chlorophyll measurement

The leaf chlorophyll content was quantified spectrophotometrically (Porra et al. 1989). Leaves were harvested and frozen in liquid nitrogen. After homogenization, chlorophyll was extracted with 80% acetone and the absorption was measured at 646.6 nm, 663.6 nm and 750 nm for the calculation of chlorophyll a and b contents.

### Tocochromanol analysis

Leaf material was frozen in liquid nitrogen and homogenized (Precellys homogenizer, PeqLab, Darmstadt, Germany). Seeds were directly homogenized. The tissue was extracted with 1 ml diethyl ether in the presence of 500 ng tocol as internal standard, followed by 300 µl 1 M KCl. After phase separation by centrifugation, the organic phase was collected, and the aqueous phase was extracted two more times with 1 ml diethyl ether each. After combining the organic phases, the diethyl ether was evaporated under nitrogen gas and the residue dissolved in hexane. Samples were dissolved in hexane. Tocopherol was measured by HPLC (Agilent 1100 system) after separation on a diol column (100 diol, 5 µm, 250 × 4 mm; Knauer, Berlin, Germany) with isocratic elution (hexane/tertiary butylmethyl ether, 96:4, v/v) with fluorescence detection (excitation, 290 nm; emission, 330 nm) (Zbierzak et al. 2010).

### Abscisic acid measurements

Leaves were frozen in liquid nitrogen and ground to a fine powder (Precellys). Phytohormones were extracted as described (Pan et al. 2010), dissolved in 0.1 ml methanol/water (1:1, v/v) with 0.1% formic acid and separated on a C18 Gemini column (5 µm, 150 × 2.0 mm, Phenomenex) using an Agilent 1260 quaternary HPLC with mobile phases as described (Pan et al. 2010). ABA was detected using a QTRAP 6500 + LC–MS/MS system (Sciex, Darmstadt, Germany) with a Turbo V ion source. The mass spectrometer was set in the negative ion mode using multiple reaction monitoring after fragmentation with a collision energy of 30 V. ABA was quantified relative to the internal standard of deuterated ABA (^2^H_6_( +)-*cis,trans*-abscisic acid, Olchemin, Olomouc, Czech Republic) (Pan et al. 2010).

### Expression analysis by qPCR

Total RNA was extracted from frozen leaves after homogenization (Precellys) using the NucleoSpin RNA Plant kit (Macherey–Nagel, Düren, Germany). Genomic DNA was removed by on-column digestion. The RNA was eluted with 50 µl of RNase-free water, and 1 µg of total RNA was used for cDNA synthesis with the First Strand cDNA Synthesis Kit (Thermo Scientific, Waltham, USA). The qPCR reactions (20 µl total) contained 10 ng cDNA, 250 nM forward and reverse primers and 4 µl my-Budget 5 × EvaGreen QPCR-Mix II (ROX) (Bio-Budget, Krefeld, Germany) (for oligonucleotides, see Table S1). qPCR was performed on the 7500 Fast Real-Time PCR system (Applied Biosystems, Foster City, USA). The qPCR protocol started with a first step at 50 ℃ for 2 min, followed by initial denaturation at 95 ℃ for 10 min, then by 40 cycles of amplification (95 ℃ for 15 s and 60 ℃ for 1 min) and a final elongation step. A dissociation curve was generated to test for specificity of the amplification. Each assay included two technical and three biological replicates and a no-template control. Gene expression data were normalized to the reference gene PP2A (protein phosphatase 2A) according to the ΔΔCt method (Livak and Schmittgen 2001). Final results represent the relative expression level of the candidate gene normalized to the expression of PP2A and referred to the expression under the respective control conditions. Data are presented on a logarithmic scale.

### Statistical analysis

Data analysis and statistical tests were performed with OriginPro9 (OriginLab Corporation, Northampton, USA). All data were normally distributed as analyzed with the Shapiro–Wilk test. Statistical significance was tested for differences between mean values from plants grown under different stress conditions for a significance level of 0.05 with analysis of variance (Fisher LSD).

## Results

### Tocochromanol accumulation in *Arabidopsis* during drought and PEG8000 treatment

**Fig. 2:**
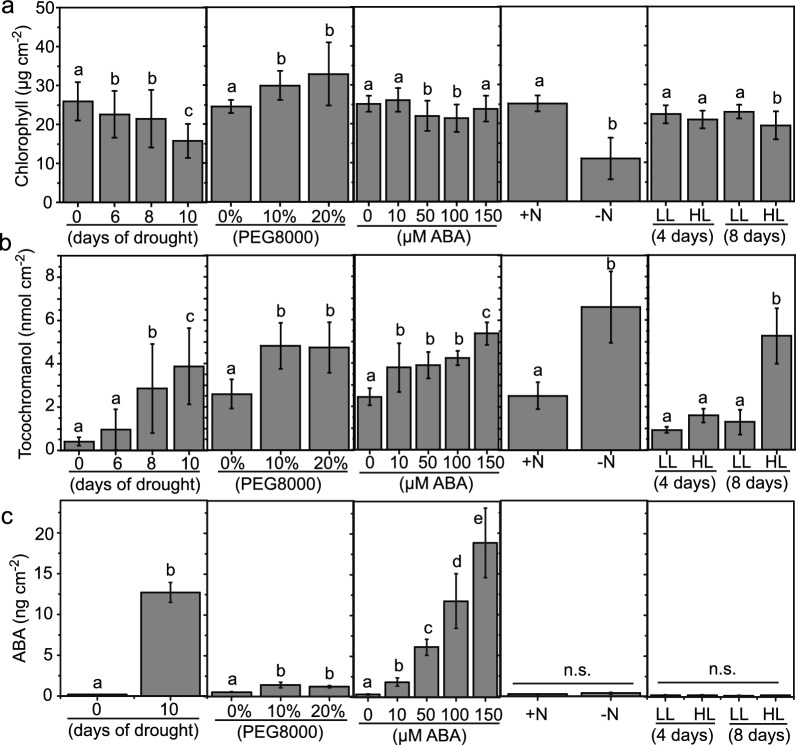
Drought stress, PEG8000, ABA treatment, N deprivation and high light differentially affect tocochromanol accumulation. *Arabidopsis* Col-0 plants were exposed to drought by withholding water for 0, 6, 8 or 10 days. For osmotic stress, plants were grown on medium containing 10% or 20% PEG8000 for 9 days. Plants were grown on medium containing 0, 50, 100 or 150 µM ABA for 9 days. Nitrogen deprivation was initiated by growing plants on synthetic –N medium for 14 days. For light stress, plants were grown for 4 or 8 days under normal (150 µmol m^−2^ s^−1^) or high light (500 µmol m^−2^ s.^−1^). **a** Chlorophyll content of Col-0 plants exposed to different stresses. **b** Tocochromanol accumulation in Col-0 plants. **c** ABA contents of *Arabidopsis* Col-0 exposed to stress. Means ± SD, *n* = 5–30. Different letters indicate significant differences. n.s., not significant; *P* < 0.05 (one-way ANOVA, Fisher LSD)

To study the impact of water deficiency on tocochromanol accumulation, we selected drought and osmotic stress caused by treatment with PEG8000 (Verslues et al. 2006; Conn et al. 2013; Frolov et al. 2017). Drought experiments can be affected by differential desiccation of the leaves, variations in soil water content, air humidity and ventilation (Abid et al. 2016). The osmotic stress in tissue culture with PEG8000 is affected by the components of the medium, because the water potential does not only depend on PEG8000, but also on the presence of MS salts and agar. The water potential was recorded as − 0.40 ± 0.05 MPa in control medium (0.3% Gelrite/without PEG8000), − 0.55 ± 0.07 in 0.6% Gelrite/10% PEG8000, and − 0.95 ± 0.06 in 0.8% Gelrite/20% PEG8000 (means ± SD; *n* = 5; all values significantly different, *p* < 0.05, one-way ANOVA, Fisher LSD). Therefore, the control medium already displays decreased water potential compared with deionized water (0.0 MPa). The low water potential of − 0.40 ± 0.05 MPa can explain the finding that the relative water content (RWC) of plants on control medium (~ 70%) was lower than that of plants growing on well-watered soil (~ 90%). Plants exposed to 10% or 20% PEG8000 showed a reduced RWC of about ~ 45% and ~ 40%, respectively (Fig. S1a). The RWC of *Arabidopsis* plants growing on soil decreased from ~ 90% in well-watered plants to ~ 70%, 50% and 30% after 6, 8 and 10 days of drought stress, respectively (Fig. S1a). Therefore, drought stress causes a stronger decrease in the RWC of plants than PEG8000 treatment. Well-watered plants displayed almost no change in chlorophyll content over 10 days. After withholding water for 8–10 days, leaves were wilted and yellowish and the chlorophyll content was decreased (Fig. S1b, Fig. S2a, Fig. 2a), in agreement with previous studies (Peisker et al. 1989; Li et al. 2006). Osmotic stress with PEG8000 caused growth retardation leading to a dark-green leaf color, in accordance with an increased chlorophyll content (Fig. 2a, Fig. S1b, Fig. S2b). The differential change in chlorophyll content can be explained by differences in growth conditions, because drought causes low soil water content and low air humidity (55%), while PEG8000 treatment causes osmotic stress in the roots, and the leaves are exposed to high air humidity (100%). The tocochromanol contents normalized to fresh weight (FW) or leaf area were increased during drought and osmotic stress, while when normalized to the dry weight (DW), tocochromanol increased only until day 8 during drought, and it even decreased on PEG8000 (Fig. S1c). Since leaves lose water during drought and osmotic stress, the FW decreases. On the other hand, the DW can increase during drought and osmotic stress, because the leaves curl and bend and accumulate lignin, together resulting in lower apparent tocochromanol contents (Moore et al. 2008; Le Gall et al. 2015). Therefore, in this study, tocochromanol were normalized to the leaf area.

### Accumulation of tocochromanols after exposure to drought, PEG8000 and ABA supplementation

To study the role of ABA during the regulation of tocopherol accumulation under different stress conditions, *Arabidopsis* plants were exposed to drought, PEG8000 or ABA treatment. ABA contents were determined in the leaves using LC–MS/MS. Drought stress for 10 days resulted in the strongest accumulation of ABA by ~ 15-fold (13 ng cm^−2^), while the ABA content was only slightly increased to 1.5 ng cm^−2^ after treatment with 10% or 20% PEG8000 (Fig. 2c). The ABA content increased by ~ 20-fold to 18 ng cm^−2^ when the plants were grown on 150 µM ABA (Fig. 2c). Therefore, drought stress and direct application of ABA resulted in strong increases in endogenous ABA levels, while PEG8000 treatment had a smaller but significant effect.

Tocochromanols were increased under all three stress conditions. The control plants of the tissue-culture-based experiments (PEG8000, ABA treatment) showed similar tocochromanol contents of ~ 2.5 nmol cm^−2^, which were higher as compared with control plants grown on soil (drought, 0.5 nmol cm^−2^), presumably because the water potential in tissue culture was decreased which caused increased tocochromanol contents compared with soil-grown plants (Fig. S1c, Fig. 2b). After 10 days of drought, the tocochromanol content increased by almost tenfold to 4 nmol cm^−2^. *Arabidopsis* plants contained ~ 5 nmol cm^−2^ tocochromanol after growth in the presence of 10% or 20% PEG8000 (Fig. 2b). ABA treatment resulted in increased tocochromanol levels in a concentration-dependent manner with an almost twofold increase to 5.5 nmol cm^−2^ at 150 µM ABA (Fig. 2b). Taken together, these results indicate that tocochromanols accumulate in *Arabidopsis* under drought and PEG8000 treatment. The results from direct ABA supplementation demonstrate that tocochromanol accumulation under these conditions is ABA-dependent.

### Tocochromanol levels increase during nitrogen deficiency and high light stress in an ABA-independent way

Next, the impact of nitrogen deficiency and high light stress on ABA and tocochromanol accumulation was studied. Plants grown under nitrogen deficiency were pale green due to chlorophyll degradation (Fig. 2a, Fig. S2c) (Gaude et al. 2007; vom Dorp et al. 2015). Under high light treatment (500 µmol m^−2^ s^−1^) for 4 or 8 days, leaves became wilted and purple, due to the accumulation of anthocyanins (Fig. S2d), and chlorophyll levels were slightly decreased (Fig. 2a). During nitrogen deprivation, the ABA content was not changed, as it remained at ~ 0.5 ng cm^−2^. Similarly, high light stress did not cause changes in ABA content, as it remained at ~ 0.2 ng cm^−2^ (Fig. 2c).

Under nitrogen deficiency, the level of tocochromanol was ~ 6.5 nmol cm^−2^ which is almost threefold higher compared with + N conditions. After 4 days of high light stress, the tocochromanol content did not significantly change, but it accumulated by fivefold to ~ 5 nmol cm^−2^ after 8 days of high light stress, in agreement with previous results (Kobayashi and DellaPenna 2008). Therefore, the two stresses, nitrogen deprivation and high light stress, which have no impact on ABA levels, also caused a strong accumulation of tocopherol, indicating that tocochromanol accumulation under these conditions was ABA-independent.

### Changes in tocochromanol composition during stress

Figure S3 shows the changes in tocochromanol composition in dependence of the stress conditions. Growth of control plants in tissue culture (PEG8000, + N/–N, ABA supplementation) resulted in higher relative amounts of PC8 compared with soil-grown plants. This increase in PC8 was presumably caused by the presence of sucrose in the medium which led to the down-regulation of photosynthesis, associated with the conversion of plastoquinone-9 into the photosynthetically inactive form of PC8 (Fig. 1).

Drought caused the strongest change in tocochromanol composition, because the proportion of α-tocopherol declined from 90 to 65%, while γ-tocopherol increased from 0.9% to 21%, and δ-tocopherol increased from ~ 0.5% to almost 9% (Fig. S3). Osmotic stress with PEG8000 resulted in the increase in the proportion of α-tocopherol at the expense of PC8, while γ-tocopherol slightly increased. After ABA application, the proportion of α-tocopherol remained almost unchanged, while PC8 decreased, and the proportions of γ-tocopherol and δ-tocopherol increased. Under nitrogen deprivation, the proportion of α-tocopherol remained similar and the amounts of β-, γ- and δ-tocopherol increased. High light treatment for 8 days resulted in the decline in the proportion of α-tocopherol from ~ 90 to ~ 75%, accompanied with the increase in the proportions of γ-tocopherol and δ-tocopherol to 13 and 4%, respectively (Fig. S3). Therefore, PC8 accumulation is associated with growth on tissue culture medium. Stress causes the relative increase in γ-tocopherol and other tocopherol forms vs. α-tocopherol, and this effect is most strongly observed during drought and ABA treatment, but also during other stresses, indicating that this shift in tocopherol composition can occur in an ABA-dependent or -independent way.

### Expression of tocopherol biosynthesis genes during abiotic stress

**Fig. 3:**
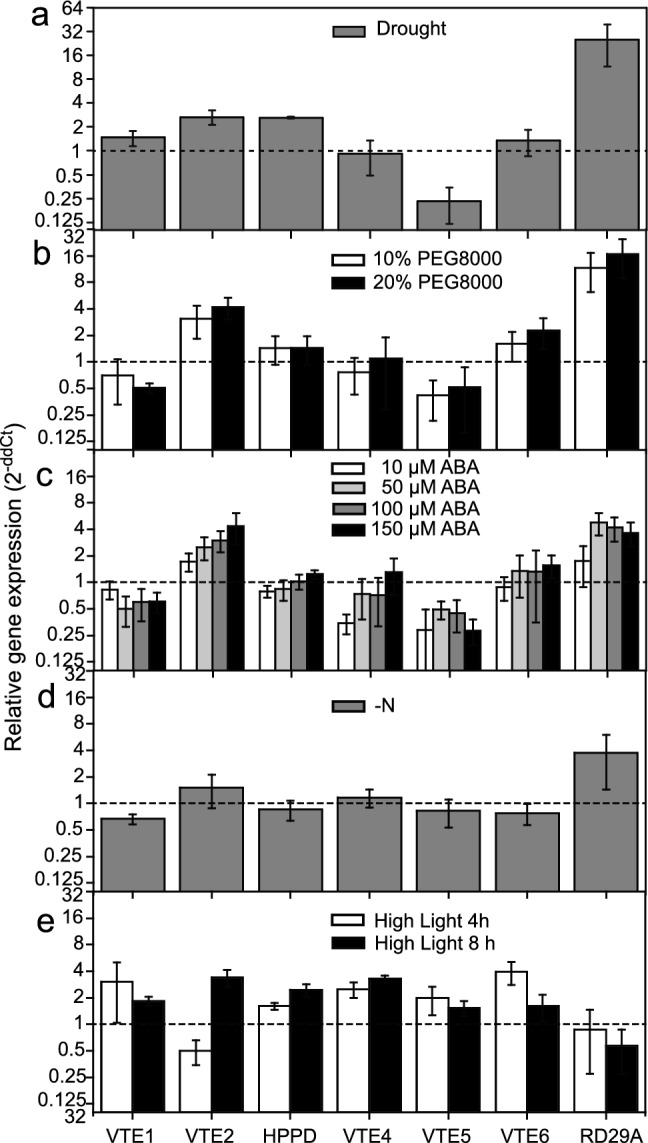
Relative expression of tocopherol biosynthetic genes in Col-0 plants exposed to abiotic stress. Expression of tocopherol biosynthesis genes (*HPPD*, p-hydroxypyruvate dioxygenase, *VTE1* tocopherol cyclase, *VTE2* homogentisate phytyltransferase, *VTE4* γ-tocopherol methyltransferase, *VTE5* phytol kinase, *VTE6* phytyl-P kinase) and of the ABA-inducible gene RD29A was recorded in leaves of Col-0 plants after **a** drought (10 days), **b** treatment with PEG8000 (9 days), **c** ABA treatment (9 days), **d** N deprivation (14 days), **e** high light (4 or 8 days). Results are presented as relative transcript abundance (2^ΔΔCt^), normalized to the expression of the reference gene *PP2A* and to the expression under the control conditions. Means ± SD, *n* = 3

The expression of tocopherol biosynthesis genes (HPPD, VTE2, VTE1, VTE4, VTE5, and VTE6) was recorded by qPCR to study the impact of different stresses on the regulation of tocopherol synthesis (Figs. 1, 3). RD29A expression is known to be upregulated by ABA, ant it was, therefore, included as control (Yamaguchi-Shinozaki and Shinozaki 1994). Under drought, RD29A expression was highest when compared with the other treatments (Fig. 3a). RD29A expression was also upregulated during PEG8000 treatment and ABA supplementation, in agreement with the finding that ABA accumulates during these three stresses (Fig. 2).

Drought resulted in the induction of expression of HPPD and VTE2, while the transcript level of VTE4 was unchanged. Similar changes in expression patterns were observed after PEG8000 treatment (Fig. 3b). VTE2 expression was also upregulated under ABA treatment, and VTE4 expression was slightly increased (Fig. 3c), suggesting that ABA contributes to the regulation of tocochromanol accumulation mostly through the regulation of VTE2 expression. VTE4 expression mostly remained unchanged, in line with the finding that VTE4 activity becomes limiting under stress conditions and ABA treatment, resulting in increased proportions of γ-tocopherol (Fig. S3).

Nitrogen deficiency showed a different pattern, because transcript abundances of tocopherol biosynthetic genes remained largely unchanged, while that of RD29A was strongly induced (Fig. 3d). Since ABA levels do not change during nitrogen deprivation (Fig. 2), the increases in RD29A expression must be ABA-independent and might be explained by the presence of cis-acting, dehydration-responsive elements (DRE) in the RD29A promoter (Yamaguchi-Shinozaki and Shinozaki 1993, 1994). In contrast, high light resulted in the induction of expression of several tocopherol biosynthesis genes (VTE1, VTE2, HPPD, VTE4 VTE5, and VTE6), while transcription of RD29A remained unchanged, in line with the finding that ABA is not involved in regulating gene expression during high light stress (Fig. 3e). These results indicate that during nitrogen deprivation, tocochromanol accumulation is independent of ABA and does not require upregulation of tocopherol biosynthetic gene expression. During high light stress, tocochromanol accumulation and tocopherol biosynthetic gene expression are upregulated in an ABA-independent way.

### Effects of mutations in ABA biosynthesis genes on tocochromanol accumulation and composition

**Fig. 4:**
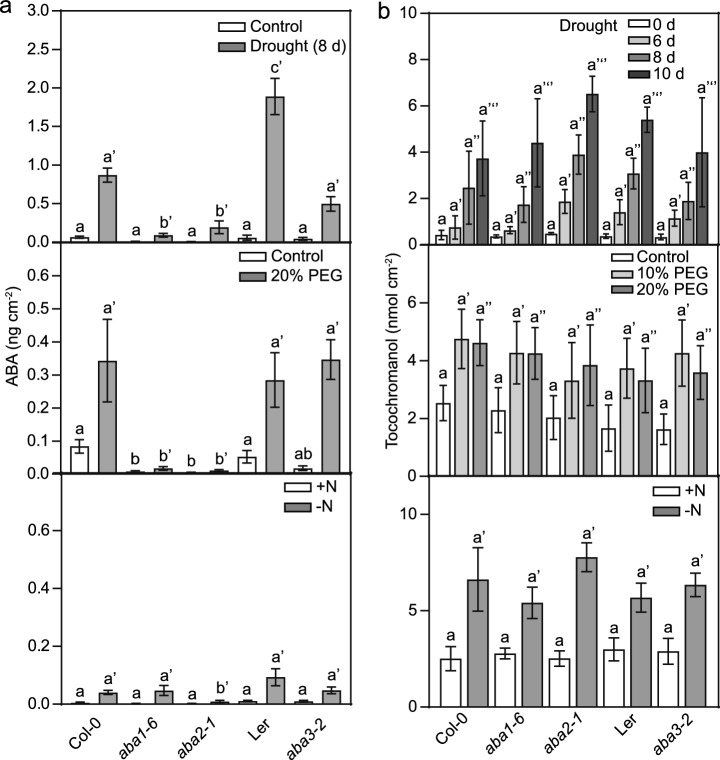
ABA and tocochromanol contents in *aba* mutants grown under drought, PEG8000 treatment, or N deprivation. ABA (**a**) and tocochromanol (**b**) contents were measured in leaves of *Arabidopsis* wild type (Col-0, Ler) and ABA-deficient mutants (*aba1-6, aba2-1* in Col-0; *aba3-2* in Ler). Plants were grown under drought by withholding water for 0, 6, 8 or 10 days; on medium containing 0%, 10% or 20% PEG8000 for 9 days; or with (+ N) and without nitrogen (–N) for 14 days. Means ± SD, *n* = 5. Different letters indicate significant differences; one-way ANOVA, Fisher LSD

Different *aba* mutants (*aba1-6, aba2-1*, and *aba3-2*) were selected to study the impact of ABA deficiency on tocochromanol accumulation. Plants were exposed to drought, PEG8000 treatment and N deprivation to stimulate tocochromanol synthesis. It is known that some *aba* mutants do not harbor null mutations, and in addition, alternative pathways can contribute to ABA synthesis bypassing the blocks in the mutant plants. We, therefore, measured ABA in *aba* plants grown under control or stress conditions by LC–MS/MS. The ABA contents in the *aba1-6*, *aba2-1* and *aba3-2* mutants during drought stress were strongly decreased compared with the wild-type lines Col-0 and Ler, respectively. After PEG8000 treatment and even more so, under nitrogen deficiency, the ABA contents in all plants were lower compared with drought stress (Figs. 2, 4a), and the differences between the wild-type and mutant lines were in part diminished. These results indicate that ABA synthesis in the *aba1-6*, *aba2-1* and *aba3-2* mutants is affected, and this becomes particularly visible under conditions when ABA accumulates under drought stress.

Figure 4b shows that the tocochromanol content in the *aba1-6, aba2-1* and *aba3-2* mutant plants increased under the three stress conditions in a similar way as compared with their corresponding wild types, Col-0 or Ler. The finding that tocochromanols, but not ABA, accumulate in the *aba1-6* and *aba2-1* mutants, demonstrates that tocochromanol increase in these mutants is ABA-independent.

**Fig. 5:**
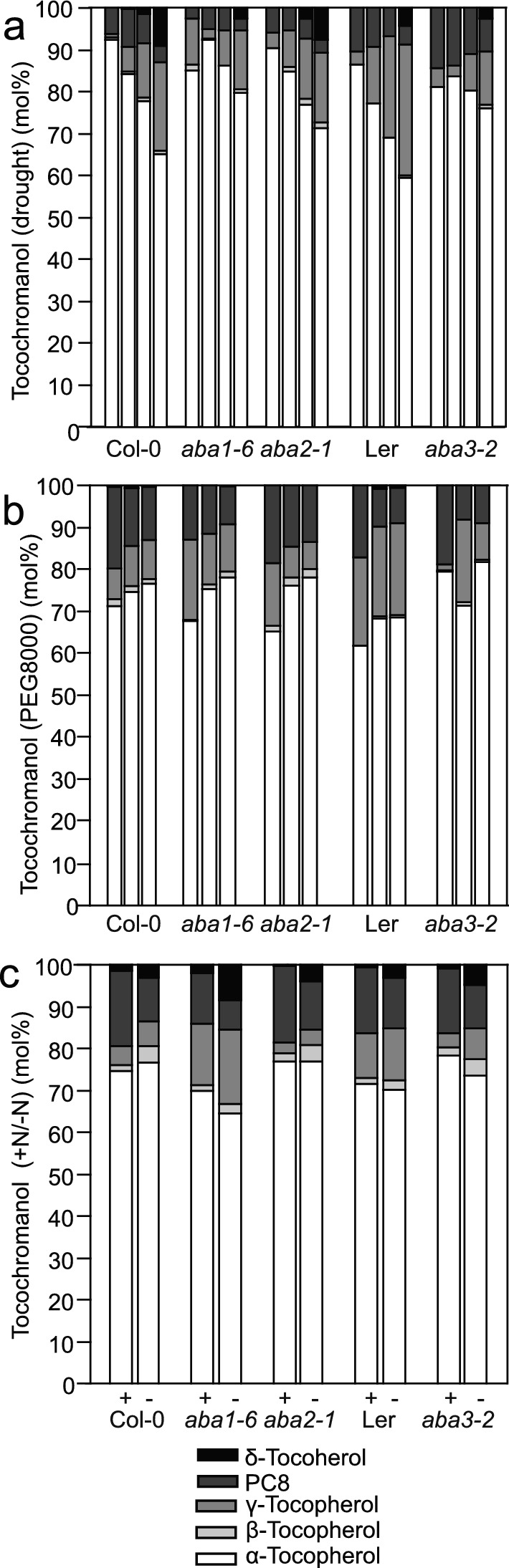
Tocopherol composition in *aba* mutants. The percent distribution of α-, β-, γ-, δ- tocopherol and plastochromanol (PC8) in ABA-deficient mutants. **a** Plants were grown under drought for 0, 6, 8 or 10 days (bars, from left to right). **b** Plants were grown on MS medium containing 0%, 10% or 20% PEG8000 (bars, from left to right) for 9 days. **c** Plants were grown under control (+ N, left bars) or N deprived (–N, right bars) conditions for 14 days. Bars represent means, *n* = 5–30

We next compared the tocochromanol composition in leaves of *aba* mutants grown under the different stress conditions. As already found for wild-type Col-0, the proportion of PC8 was elevated in all plants growing on tissue culture medium (PEG8000, + N/– N) compared with soil (drought) (Fig. S3, Fig. 5). The decrease in the proportion of α-tocopherol accompanied with the increase in δ-tocopherol and γ-tocopherol in the wild-type lines (Col-0, Ler) during drought was suppressed in the *aba1-6*, *aba2-4*, and *aba3-2* mutants (Fig. S3, 5a), but this effect was less obvious during PEG8000 treatment or nitrogen deprivation.

High amounts of tocochromanols were found in seeds of the *aba* mutants, similar to the Col-0 or Ler wild types (Fig. S4a). In addition, the tocochromanol composition in the *aba* mutant seeds was similar to Col-0 and Ler, with mostly γ-tocopherol (85–90%), and lower proportions of the other tocochromanols, in agreement with previous results (Shintani and DellaPenna 1998) (Fig. S4b). Therefore, the *aba* mutants did not display any changes in the tocochromanol content or composition in the seeds.

## Discussion

The analysis of the regulatory network between ABA and tocochromanol synthesis is crucial to understand the role of tocochromanol during environmental stresses. Five stress conditions were employed in this study: (i) drought, (ii) PEG8000-induced osmotic stress, (iii) application of ABA, (iv) N deprivation, and (v) high light. All stress treatments resulted in the accumulation of tocochromanols, but only some of them resulted in the increase in ABA content, or in the induction of expression of tocopherol biosynthesis genes.

### Increase in tocochromanol contents during stresses associated with ABA accumulation

ABA increases strongly during drought and after ABA treatment, and it is also elevated under osmotic stress (PEG8000). Under these conditions, the expression of the tocopherol synthesis gene *VTE2*, and to a minor extent, of *HPPD*, was induced. It remains unclear whether ABA accumulation during drought and osmotic stress is causal for the increase in tocochromanol. The results from ABA treatment demonstrate that the tocochromanol accumulation under these conditions is regulated by ABA-dependent induction of *VTE2* and *HPPD* expression. VTE2 is involved in the condensation of homogentisate with phytyl-PP, while HPPD produces the precursor homogentisate (Fig. 1) (Collakova and DellaPenna 2003b). An increased availability of homogentisate and 2-methyl-6-phytyl-benzoquinol (MPBQ) contributes to tocopherol accumulation under stress. ABA-responsive elements (ABREs) were previously identified in the promoter regions of the tocopherol biosynthesis genes OsHPPD, OsγTMT (OsVTE4) and OsMPBQMT1 (OsVTE3) of rice, and transcript abundances of these genes were increased during dehydration stress (Chaudhary and Khurana 2009). A positive correlation between ABA, tocopherol gene expression (HPPD, VTE2, VTE1, and VTE4) and tocochromanol contents was revealed during drought and ABA treatment (Yamaguchi-Shinozaki and Shinozaki 1994; Ghassemian et al. 2008; Fleta-Soriano and Munné-Bosch 2017).

### ABA-independent accumulation of tocochromanols in ABA-deficient mutants

The selected *aba* mutants displayed minor differences in ABA levels compared with the wild types Col-0 or Ler under control conditions or under nitrogen deprivation. However, the mutants showed strongly decreased ABA levels under drought and PEG8000 treatment, when ABA accumulates in the wild type (Fig. 4a). The residual amount of ABA observed under control and stress conditions in the *aba* mutants might originate from leaky mutations (*aba1-6,* Gly160Ser; *aba2-1,* Ser264Asn) (Léon-Kloosterziel et al. 1996; Niyogi et al. 1998; Cheng et al. 2002; Barrero et al. 2005), or by the presence of paralogous enzymes (*aba3-2*, Leu387stop) (Xiong et al. 2001). The tocochromanol contents of the *aba* mutants were not different from the corresponding wild types after exposure to stress (Fig. 4b). Therefore, the accumulation of tocochromanol in the *aba* mutants under stress is mostly independent of ABA.

### Tocochromanol levels increase during stresses which are independent of ABA

In contrast to drought, treatment with PEG8000 or ABA, the other two stress conditions of nitrogen deprivation or high light stress did not cause ABA accumulation, but still resulted in the increase in tocochromanol levels. The expression of the tocopherol biosynthesis genes remained mostly unchanged during N deficiency, posing the question how tocopherol synthesis is regulated under these conditions. A strong decrease in chlorophyll levels was observed in the leaves during nitrogen deficiency (Fig. 2a). A correlation between chlorophyll degradation and tocopherol accumulation during leaf senescence has previously been reported (Rise et al. 1989). Phytol, released during chlorophyll breakdown, is phosphorylated by phytol kinase activities (VTE5, FOLK) and phytyl-P kinase resulting in the production of phytyl-PP, the substrate for 2,3-dimethyl-5-phytyl-benzoquinol (DMPBQ) synthesis by VTE2 (Valentin et al. 2006; vom Dorp et al. 2015; Romer et al. 2024). Therefore, the increase in tocopherol levels under nitrogen deprivation might in part be regulated by the increased availability of the precursor phytol which is released from chlorophyll degradation.

High light treatment also did not affect ABA contents, but at the same time, it resulted in the increased expression of tocopherol biosynthesis genes (VTE1, VTE2, HPPD, VTE4, VTE5, and VTE6) (Figs. 2, 3d). ABA levels were not increased during high light stress in line with the lack of induction of RD29A expression, and therefore, the accumulation of tocochromanols and the induction of expression of tocopherol biosynthesis genes are independent from ABA.

### Changes in tocochromanol composition during stress

The increase in tocochromanol levels under stress was paralleled by a decrease in the α-tocopherol proportion and an increase in the proportions of δ-tocopherol and γ-tocopherol. Therefore, the ratios of γ-tocopherol or δ-tocopherol to α-tocopherol increase under all stress conditions, suggesting that the γ-tocopherol methyltransferase (VTE4) activity becomes limiting (Fig. 1, Fig. S3). This result is in line with the finding that VTE4 expression remains mostly unchanged during stress (Fig. 3). The tocochromanol composition in the *aba* mutants exposed to drought was different, because the decrease in the proportion of α-tocopherol and the increase in the proportion γ-tocopherol observed in the wild types were suppressed (Fig. 5). A similar tendency was observed for PEG8000 treatment. Drought stress is accompanied with a strong accumulation of ABA (Fig. 2c), in contrast to PEG8000 treatment which causes a much lower increase in ABA (Fig. 5). These results suggest that the compromised ABA synthesis in the *aba* mutants during drought is accompanied with the increased conversion of γ-tocopherol to α-tocopherol.

### Regulation of tocochromanol synthesis during different stresses

The measurements of ABA and expression analysis of tocopherol biosynthetic genes during stress indicate that tocochromanol accumulation is regulated by different signaling pathways. Drought, osmotic stress and ABA supplementation result in ABA accumulation and upregulation of expression of key tocopherol genes. The increase in gene expression after addition of ABA is very likely mediated through ABA-dependent responses. The finding that tocopherol still increases in the *aba1-6*, *aba2-1* and *aba3-2* mutants during drought and osmotic stress suggests that tocopherol synthesis under these conditions is largely ABA-independent, or that an ABA-independent pathway becomes predominant in the mutants where regulation of tocopherol synthesis through ABA is abolished. Under nitrogen deprivation, ABA does not accumulate and tocopherol biosynthetic gene expression is unchanged. The increase in tocochromanols under nitrogen deprivation might be explained by the release of the tocopherol precursor phytol from chlorophyll degradation. During high light treatment, expression of tocopherol biosynthetic genes and accumulation of tocochromanol are regulated independently of ABA.

Therefore, both ABA-dependent and ABA-independent signaling pathways contribute to the induction of gene expression and the accumulation of tocochromanol under the different stress conditions. The ABA-independent regulation of tocopherol synthesis might be due to other signaling pathways including alternative phytohormones. Treatment of cannabis leaves with gibberellic acid (GA) led to increased amounts of α-tocopherol (Mansouri et al. 2009). Moreover, the seeds of two *Arabidopsis* salicylic acid-(SA) deficient mutants, *NahG* and *sid2,* contained more tocopherol than the wild type (Abreu and Munné-Bosch 2009). Jasmonic acid (JA) is involved in the regulation of tyrosine aminotransferase (TAT), which catalyzes the transamination of tyrosine to p-hydroxyphenylpyruvate (HPP), one of the precursors for tocopherol biosynthesis (Fig. 1) (Sandorf and Holländer-Czytko 2002). In principle, it is possible that multiple phytohormones might mediate tocopherol biosynthesis by additive, synergistic or antagonistic activities.

## Supplementary Information

Below is the link to the electronic supplementary material.

Supplementary file1 (DOCX 15 KB)

Supplementary file2 (PDF 2029 KB)

## Data Availability

All data underlying this article are included in this published article and its supplementary information files.

## Abbreviations

ABA: Abscisic acid
DMPBQ: 2,3-Dimethyl-5-phytyl-benzoquinol
HGA: Homogentisate
HPPD: p-Hydroxyphenylpyruvate dioxygenase
MPBQ: 2-Methyl-6-phytyl-benzoquinol
MS: Murashige and Skoog
MSBQ: 2-Methyl-6-solanesyl-benzoquinol
PC8: Plastochromanol-8
PEG: Polyethylene glycol
Phytyl-P: Phytyl-phosphate
Phytyl-PP: Phytyl-diphosphate
Solanesyl-PP: Solanesyl-diphosphate
PQ-9: Plastoquinol-9
VTE1: Tocopherol cyclase
VTE2: Homogentisate phytyltransferase
VTE3: 2-Methyl-6-phytyl-benzoquinol methyltransferase
VTE4: γ-Tocopherol methyltransferase
VTE5: Phytol kinase
VTE6: Phytyl-phosphate kinase
