# Supplementary material for: Regulation of tocopherol (vitamin E) biosynthesis by abscisic acid-dependent and -independent pathways during abiotic stress in Arabidopsis
Source: Planta. 2025 Mar 20;261(4):94. doi: 10.1007/s00425-025-04670-9 (PMC11926002; doi:10.1007/s00425-025-04670-9)
Supplement: Supplementary file 1 — Supplementary file1 (DOCX 15 KB) [file 425_2025_4670_MOESM1_ESM.docx]

**Regulation of Tocopherol (Vitamin E) biosynthesis by abscisic acid-dependent and independent pathways during abiotic stress in Arabidopsis**

Victoria Kreszies, Katharina Gutbrod and Peter Dörmann^1^

**Supplementary Tables**

**Table S1.** Synthetic Oligonucleotides for qPCR Analyses

Oligonucleotides were ordered from IDT Genomics (Leuven, Belgium).

| Oligo-nucleotide | Comment | AGI Code | | Sequence 5’ - 3’ |
| --- | --- | --- | --- | --- |
| bn2109 | VTE6-qPCR-fw | At1g78620 | ATTGGTTCAAGTGCTGCTGG | |
| bn2110 | VTE6-qPCR-rev | At1g78620 | AGCTCCCTCTGTTCCTCTTG | |
| bn2111 | VTE1-qPCR-fw | At4g32770 | ATTTGCGATGATGGCCGTAC | |
| bn2112 | VTE1-qPCR-rev | At4g32770 | AACTCAAACCTTTCACCGCC | |
| bn2113 | VTE2-qPCR-fw | At2g18950 | CCCTATCTTCCATTGGCATCA | |
| bn2114 | VTE2-qPCR-rev | At2g18950 | CAATCCACCCAAGCCAGAA | |
| bn2115 | HPPD-qPCR-fw | At1g06570 | AGGGACGTTGCTTCAAATCTTC | |
| bn2116 | HPPD-qPCR-rev | At1g06570 | CAAAACCACCACATCCTCCA | |
| bn2119 | VTE4-qPCR-fw | At1g64970 | GCTTTTATGACCCTGATTCTTCTGT | |
| bn2120 | VTE4-qPCR-rev | At1g64970 | CACACCCAACATCCACTACTTTCT | |
| bn2125 | VTE5-qPCR-fw | At5g04490 | ATGGCAGCAACCTTACCTCT | |
| bn2126 | VTE5-qPCR-rev | At5g04490 | ACGTCATGCAACAGCGAATT | |
| bn2903 | PP2A-qPCR-fw | At1g13320 | TAACGTGGCCAAAATGATGC | |
| bn2904 | PP2A-qPCR-rev | At1g13320 | GTTCTCCACAACCGCTTGGT | |
| bn2963 | RD29A-qPCR-fw | At5g52310 | GTTACTGATCCCACCAAAGAAGA | |
| bn2964 | RD29A-qPCR-rev | At5g52310 | GGAGACTCATCAGTCACTTCCA | |
