# Supplementary material for: Regulation of tocopherol (vitamin E) biosynthesis by abscisic acid-dependent and -independent pathways during abiotic stress in Arabidopsis
Source: Planta. 2025 Mar 20;261(4):94. doi: 10.1007/s00425-025-04670-9 (PMC11926002; doi:10.1007/s00425-025-04670-9)
Supplement: Supplementary file 2 — Supplementary file2 (PDF 2029 KB) [file 425_2025_4670_MOESM2_ESM.pdf]

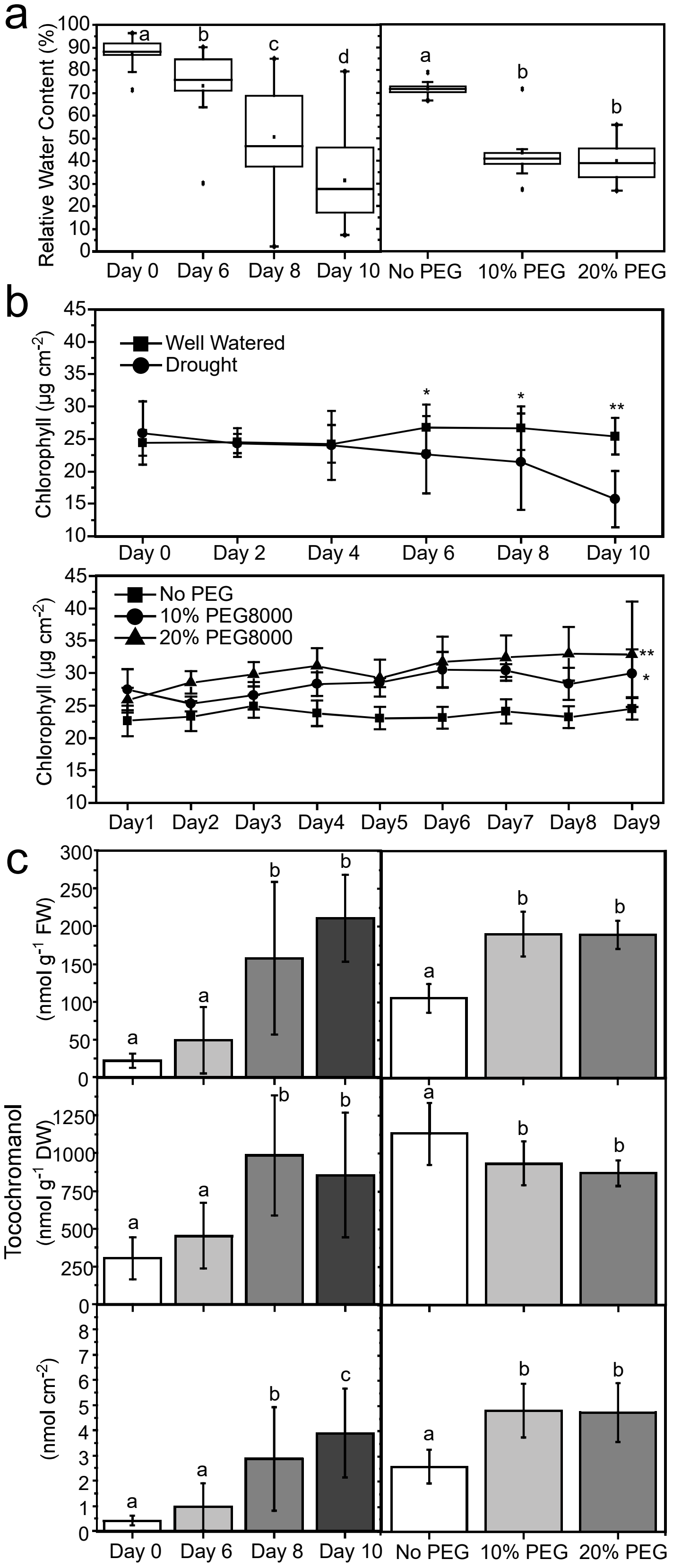

Fig. S1 Drought and PEG8000 stress have different effects on relative water content, chlorophyll and tocopherol levels.

Col-0 plants were grown on soil under drought (left panels) for up to 10 days, or on medium with 0%, 10% or 20% of PEG8000 (right panels) for 9 days. **a** Relative water content (RWC). The boxes show 25 to 75 percentiles, the squares depict the means and the whiskers indicate the outliers (n=20). Different letters indicate significant differences. P < 0.05, one-way ANOVA, Fisher LSD.
b Chlorophyll content. Asterisks indicate significant differences between means of control and stress treatments. *, P < 0.05; ** P < 0.01, one-way ANOVA, Fisher LSD. (C) Tocochromanol accumulation. Tocochromanol amounts were normalized to the fresh weight (FW, top), dry weight (DW, middle) or the leaf area (bottom). Means ± SD, n=20-35. Letters indicate differences. P < 0.05, one-way ANOVA, Fisher LSD.

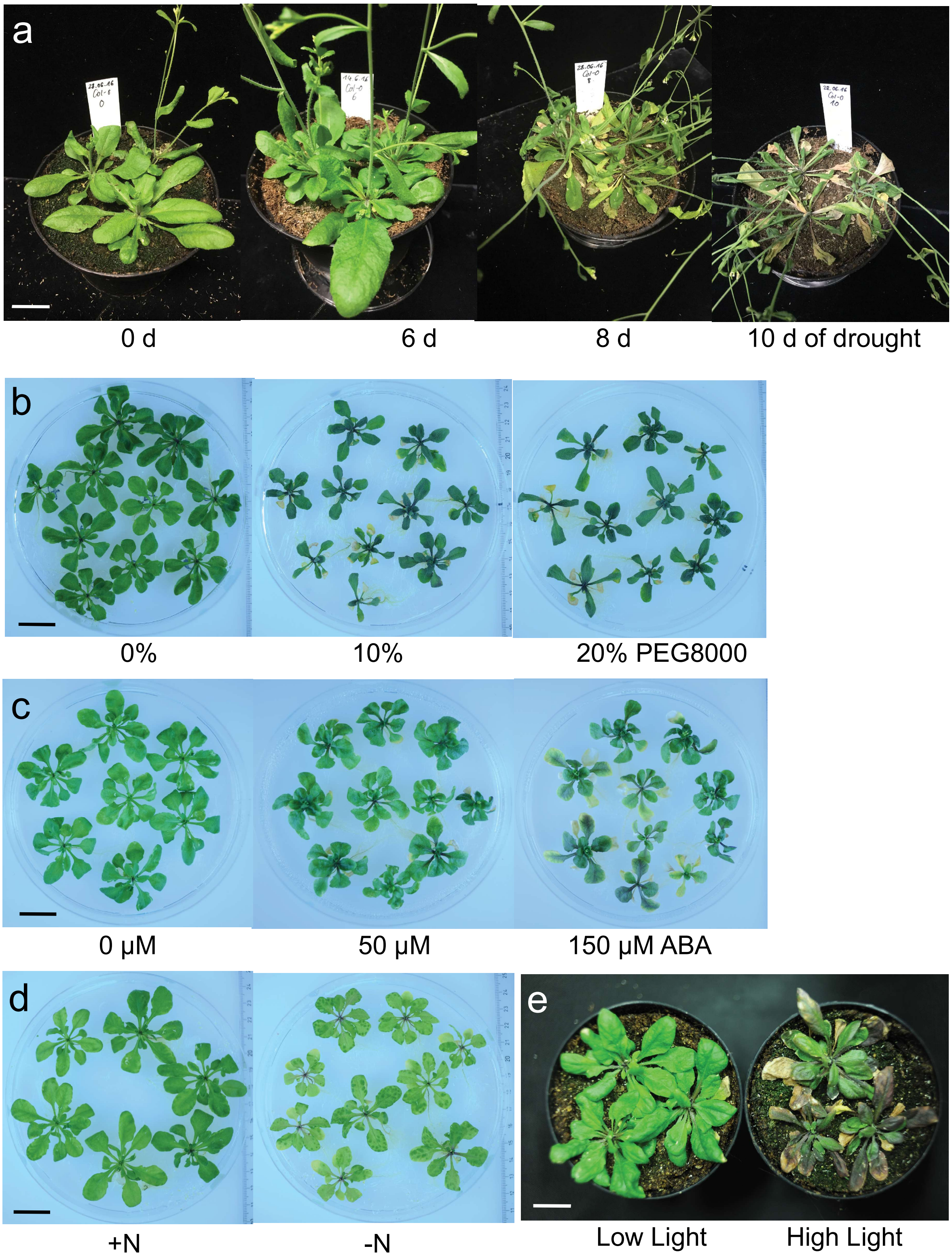

Fig. S2 Arabidopsis Col-0 plants exposed to different abiotic stress conditions.
a Plants were grown on soil and water was withheld for 0, 6, 8 or 10 days. **b** Plants were grown on MS medium containing 0, 10 or 20% PEG8000 (w/v) for 9 days. **c** Plants grown on MS medium with 0, 50 or 150 µM ABA for 9 days. **d** Col-0 plants grown under control (+N) and N deprived (-N) conditions for 14 days. **e** Col-0 plants grown on soil under low light (150 µmol s^{-1} m^{-2}) or high light (500 µmol s^{-1} m^{-2}) for 8 days. Bars = 2 cm.

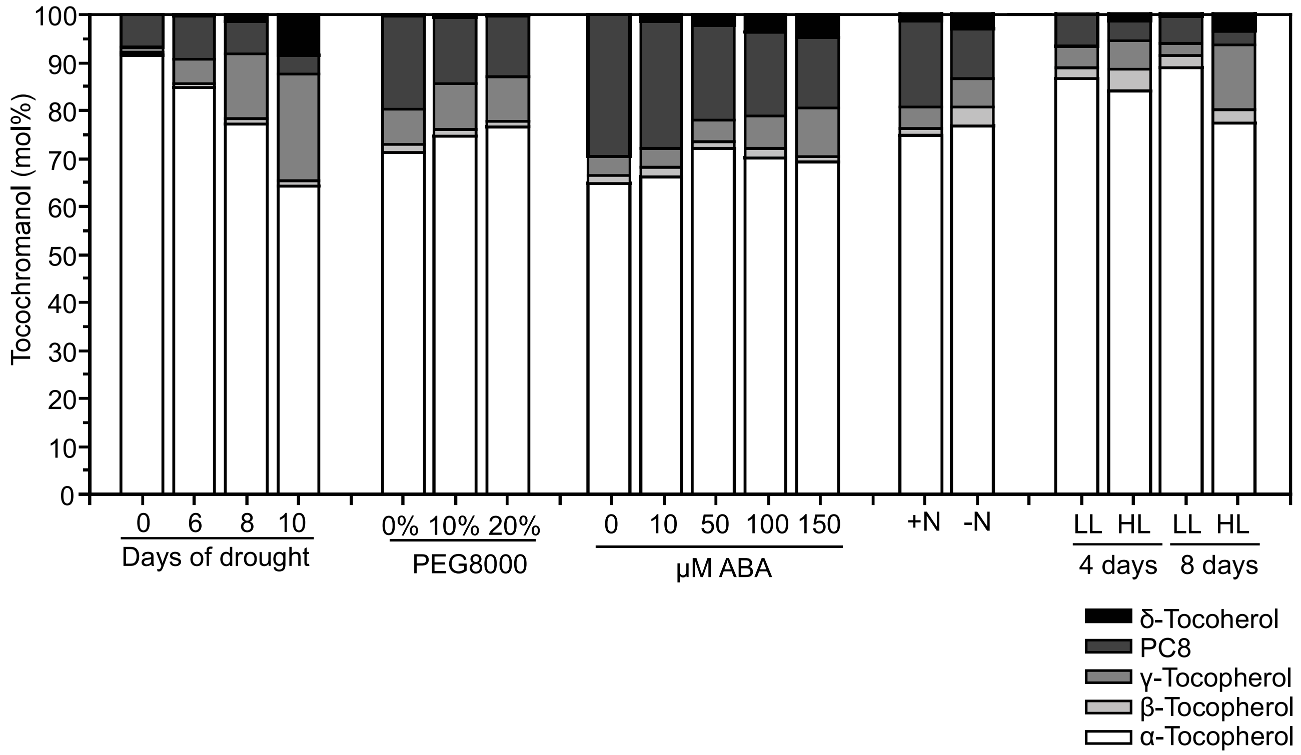

Fig. S3 Tocochromanol composition of Col-0 plants exposed to abiotic stresses. Distribution of α-, β-, γ-, δ- tocopherol and plastochromanol-8 (PC-8) in leaves of Col-0 plants after drought stress (0, 6, 8 or 10 days), PEG8000 treatment (0, 10, 20 % PEG8000; 9 days), treatment with ABA (0, 10, 50, 100 or 150 µM; 9 days), N deprivation (+N, -N; 14 days), or high light stress (low light, 150 µmol s^{-1} m^{-2}; high light, 500 µmol s^{-1} m^{-2}; 4 or 8 days). Bars represent means; n=5-3.

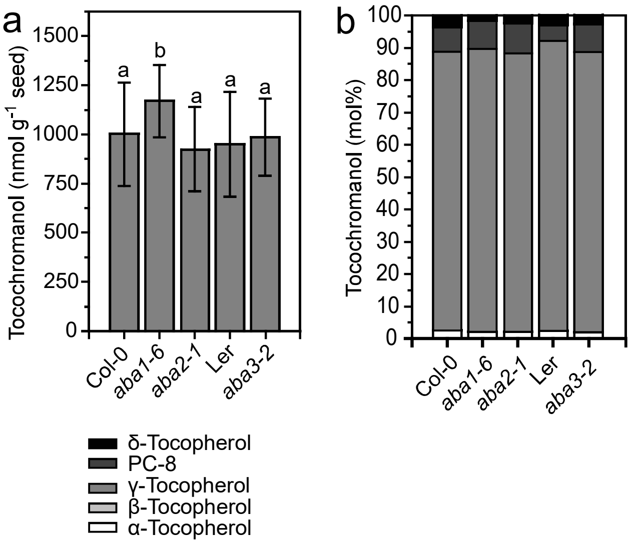

Fig. S4 Tocochromanol contents and composition in seeds of *aba* mutants
Tocochromanols were measured in seeds of *aba* mutants. **a** Tocochromanol content. **b** Tocochromanol composition. Means ± SD; n=6. One replicate equivalent to ~20 mg seeds. Different letters indicate significant differences. $P < 0.05$ (one-way ANOVA, Fisher LSD).
